# The Semi-Supervised Strategy of Machine Learning on the Gene Family Diversity to Unravel Resveratrol Synthesis

**DOI:** 10.3390/plants10102058

**Published:** 2021-09-29

**Authors:** Jun-Tae Song, Dong-U Woo, Yejin Lee, Sung-Hoon Choi, Yang-Jae Kang

**Affiliations:** 1 Division of Bio & Medical Bigdata Department (BK4 Program), Gyeongsang National University, Jinju 52828, Korea; sjt0332@gnu.ac.kr (J.-T.S.); dongu7610@naver.com (D.-U.W.); biezz0210@gmail.com (Y.L.); sungh716@gmail.com (S.-H.C.); 2 Division of Life Science Department, Gyeongsang National University, Jinju 52828, Korea

**Keywords:** resveratrol synthesis, machine learning, gene family expansion, synthetic biotechnology

## Abstract

Resveratrol is a phytochemical with medicinal benefits, being well-known for its presence in wine. Plants develop resveratrol in response to stresses such as pathogen infection, UV radiation, and other mechanical stress. The recent publications of genomic sequences of resveratrol-producing plants such as grape, peanut, and eucalyptus can expand our molecular understanding of resveratrol synthesis. Based on a gene family count matrix of *Viridiplantae* members, we uncovered important gene families that are common in resveratrol-producing plants. These gene families could be prospective candidates for improving the efficiency of synthetic biotechnology-based artificial resveratrol manufacturing.

## 1. Introduction

Resveratrol is a pharmaceutically beneficial phytochemical, being well-known for its presence in wine. It is a member of the stilbene family and commonly exists in the cis or trans type [1]. They are reported to have potential anticancer abilities, including their involvement in tumor initiation, promotion, and progression [2]. Moreover, it has been reported to have protective effects on inflammation, neuro-, and cardiovascular disease as well as on the immune system [3,4,5], while its impact on human health is debatable [6,7]. The demand for supplements and functional foods is growing as a result of the phytochemical’s benefits on human health, and resveratrol-related foods and supplements have created a huge market. Resveratrol has been found in grapes, peanuts, eucalyptus, etc. Stresses such as pathogen infection, UV radiation, and other mechanical stresses cause the plants to accumulate resveratrol to protect themselves [8]. Understanding the molecular mechanism of resveratrol synthesis in plants would be useful for genomic engineering to optimize resveratrol production in the absence of additional stress treatments. Further, it can be applied to synthetic biology for producing resveratrol in *E. coli* [9]. Currently, several gene components in the resveratrol synthesis pathway have been suggested and registered in the KEGG database [10] as the “Stilbenoid, diarylheptanoid and gingerol biosynthesis” pathway (map00945). Stilbene synthase (K13232) is an enzyme in the reaction producing resveratrol and its gene family forms a huge cluster in the Viridiplantae clade. Molecular knowledge of resveratrol synthesis can be expanded by the construction of genome sequences of resveratrol producing plants such as grape, peanut, and eucalyptus [11,12,13]. Since resveratrol is a secondary metabolite that is affected by environmental factors, the responsible genomic components in resveratrol-producing plants would have gone through a similar evolutionary process. The evolutionary genomic traces of plants can be extracted by a bioinformatic analysis based on reference genome sequences. Moreover, the evolution of environmental stress-related genes can be expected to be very fast and versatile because the environments that many plant species encounter and adapt to are harsh and dynamic. Therefore, the gene family evolution traces may show their history of environmental adaptation indirectly and gene families with adaptation signals would be beneficial to understand the phytochemical production and their pathway. Here, we discover key gene families that are prevalent in resveratrol-producing plants based on a gene family count matrix of Viridiplantae members, Their evolutions were highly diverse based on their protein sequences suggesting their fast evolution for survival. Listed gene families that are prevalent in resveratrol production would be informative as candidate genes for resveratrol synthesis. Furthermore, they can be supportive genes showing enhancing effects on resveratrol synthetic pathways. Therefore, these gene families would be potential candidates that would increase the efficiency of artificial resveratrol synthesis by synthetic biotechnology.

## 2. Results

### 2.1. Gene Family Evolution of the Resveratrol Synthesis Pathway

**Figure 1:**
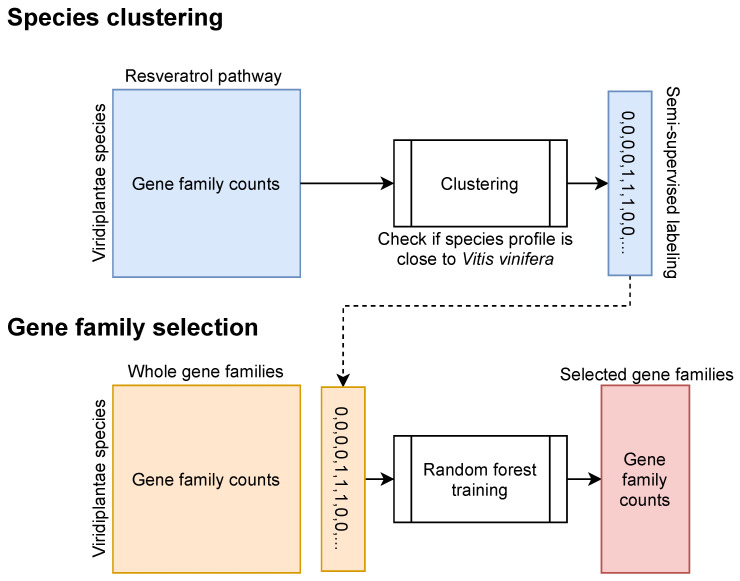
A semi-supervised machine learning strategy to investigate key resveratrol synthesis gene families.

**Figure 2:**
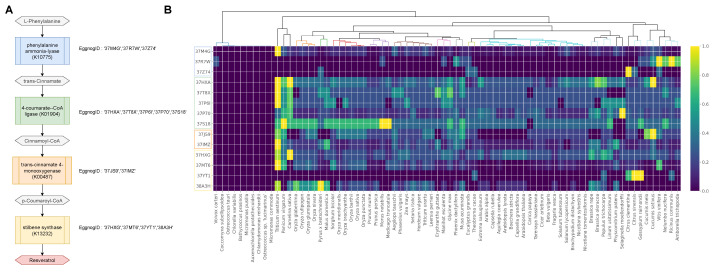
Resveratrol pathway gene families in *Viridiplantae* species. (**A**) Resveratrol synthesis pathway with corresponding Eggnog cluster IDs. (**B**) Heatmap display of gene families’ member counts in the pathway.

The functional evolution of genes involved in the development of defensive secondary metabolites can take place in various ways to cope with environmental changes. As a defensive secondary metabolite, resveratrol production can be triggered by pathogen infection and drought treatment [1]. However, not all existing plants generate resveratrol, and only a few species have been shown to produce resveratrol in significant amounts. As a result, we attempted to evaluate the evolutionary closeness of plant species by using the gene family profile of the known resveratrol synthesis pathway (Figure 1). We identified the gene families of the known key genes in the resveratrol synthesis pathway in the Viridiplantae clade and examined the presence/absence and copy number of the gene families in each species to understand the evolution (Figure 2). We surveyed the resveratrol synthesis pathway based on the KEGG database [10]. Resveratrol synthesis key genes were found in the pathways of “Stilbenoid, diarylheptanoid and gingerol biosynthesis” (map00945) and “Biosynthesis of phenylpropanoids” (map01061). Figure 2A shows the enzymes and intermediate compounds in the pathway toward resveratrol synthesis, including phenylalanine ammonia-lyase (K10775), 4-coumarate-CoA ligase (K01904), trans-cinnamate 4-monooxygenase (K00487), and stilbene synthase (K13232).

Based on the documented genes for each KEGG orthology ID, we retrieved the Eggnog ID using the Emapper script provided by the Eggnog [14]. We assigned multiple Eggnog IDs to each KEGG Ortholog ID and counted the copy number (Figure 2B and Figure S1). The copy number heatmap showed the overall copy number profile of the key gene families in the resveratrol synthesis pathway. The absence of these gene families in green algae such as *Micromonas*, *Chlamydomonas*, *Ostreococcus*, *Auxenochlorella*, *Chlorella*, *Coccomyxa*, and *Volvox* suggested that the resveratrol synthesis pathway evolved after land plants appeared. Supporting the hypothesis, *Physcomitrella* (mosses) and *Selaginella* (lycopod), for example, have the gene families despite having different copy numbers than the other land plants.

### 2.2. Mining Key Gene Families in the Resveratrol Synthesis Pathway

**Figure 3:**
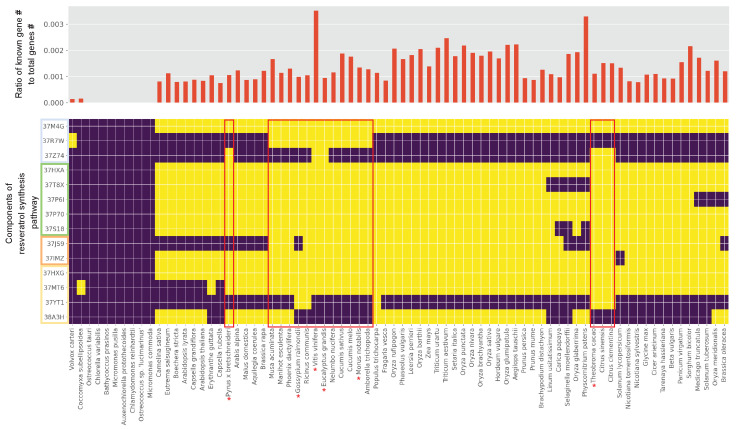
Presence or absence of resveratrol pathway member. The existence of the gene family is represented by the yellow box, while the lack is shown by the purple box. The ratio of resveratrol pathway gene number to total gene number can be seen in the top panel.

**Figure 4:**
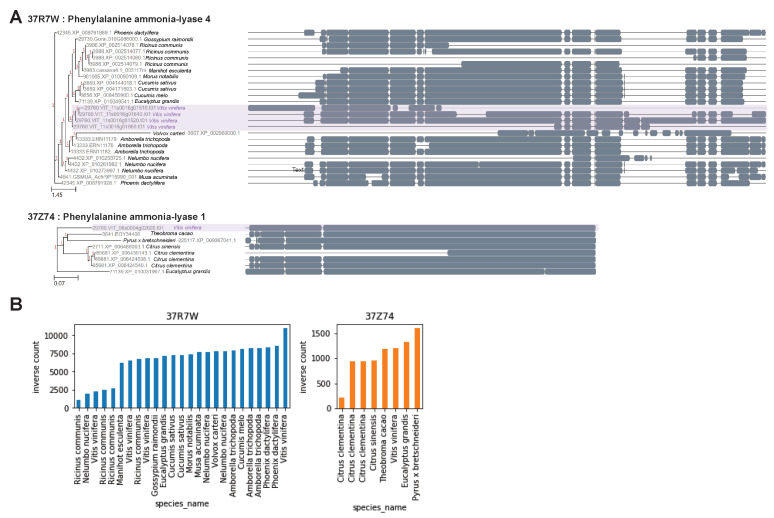
The visualization of protein shows distinct evolutionary traces of PAL4 in *V. vinifera* compared to other land plants. (**A**) The dendrogram among species and their conserved domains by protein alignment of the gene families. (**B**) Bar plot showing the uniqueness of amino acids in the gene families.

*Vitis vinifera*, especially, showed a highly increased copy number in phenylalanine ammonia-lyase (K10775) and stilbene synthase (K13232). The notable gene family that distinguished the species was phenylalanine ammonia-lyase (K10775). The presence and absence of the gene family showed apparent clustering of the species (Figure 3). Among the phenylalanine ammonia-lyase (PAL) gene families, the 37M4G cluster (PAL, PAL2, and PAL7 in *V. vinifera*) was commonly present in the land plants; however, 37R7W (PAL4 in *V. vinifera*) and 37Z74 clusters (PAL1 in *V. vinifera*) existed in a few plant species. We surveyed whether each species had at least two gene clusters of PAL. Several species within the criteria, including *Pyrus* [15], *Gossypium* [16], *Morus* [17], *Theobroma* [18], *Eucalyptus*, and *Vitis* [19], were reported as resveratrol-producing plants. *V. vinifera* showed the highest ratio of key gene counts to its total number of genes, possibly reflecting its high resveratrol content (Figure 3). Supporting the possibility, reported resveratrol contents [20] are increasing along with the ratio of key gene counts to its total number of genes (Figure S2). Notably, *Physcomitrium patens* also showed a high ratio such as *V. vinifera*; however, its resveratrol content is currently unknown. Especially, the 37MT6 cluster of the stilbene synthase gene family was highly duplicated in *V. vinifera*, resulting in 49 inparalogs (Figure S1, Table S1). Moreover, the protein alignments of 37R7W and 37Z74 gene clusters revealed a distinct evolution of protein sequences in *V. vinifera* than other plant species (Figure 4A). To quantify the distinct evolution of *V. vinifera*, we counted the amino acid at a position of a species against the amino acids at the position of all species in the protein alignments. We ignored the gap (‘-’) for the calculation. The number of species in the alignment was subtracted by the sum of the counts of each species in the alignment (Figure 4B). Hence, the higher value would represent a low frequency of amino acids among the species in the alignment. Based on the inverse count plot of 37R7W, we could quantitatively find that a protein of *V. vinifera* contained many rare amino acids compared to others. For the 37Z74, *Pyrus*, *Eucalyptus*, *Vitis*, and *Theobroma* showed proteins that contained rare amino acids, and they were all reported to produce resveratrol. These findings indicated that the predominance of resveratrol synthesis in *V. vinifera* may be due to the copy number increase and diversification of the gene families.

### 2.3. Additional Gene Family Evolution for Resveratrol Synthesis by Machine Learning

**Figure 5:**
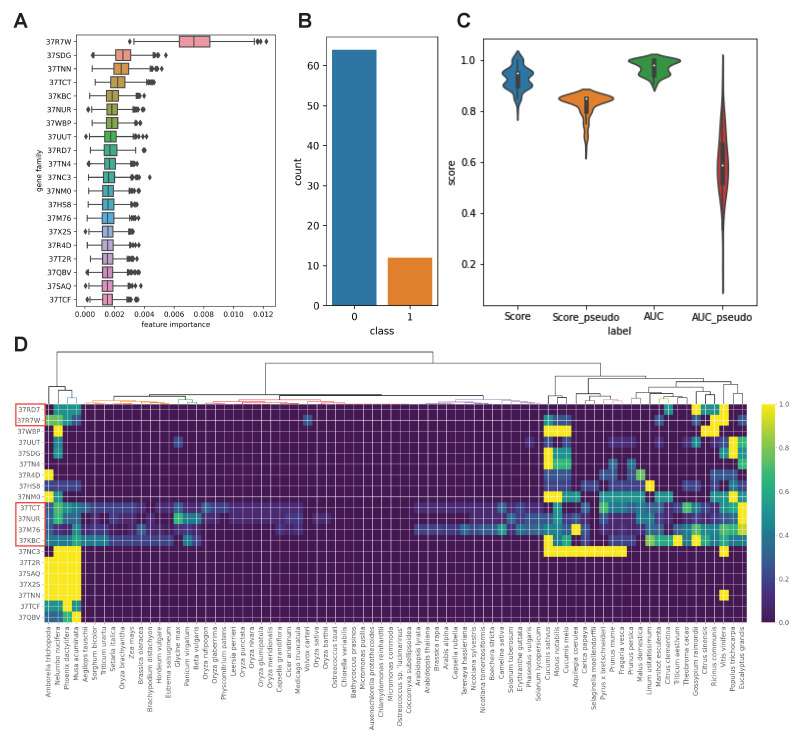
Random forest based gene family selection. (**A**) Feature importance distribution from 1000 times iterative training of random forest. (**B**) Count plot showing biased label classes of our dataset. (**C**) Accuracy score and AUC comparison between true model and pseudo model with random labels. (**D**) Heatmap display of the selected gene families’ member counts. The counts of gene family members were normalized to allow comparisons across species. The gene families associated with the species that produce resveratrol are highlighted in the red box.

The PAL and stilbene synthase gene families may have evolved dynamically in the plant species, resulting in a quantitatively different resveratrol synthesis. To further understand the evolution of the resveratrol synthesis, we checked if there were co-evolving gene families based on the whole set of the eggnog cluster count matrix (Table S1). We assigned a 1 to species with multiple PAL gene families and a 0 to species without or with only one PAL gene family as classification labels (Figure 1). We used supervised machine learning algorithms to extract important gene families involved in resveratrol synthesis based on the labels. A ridge regression classifier was trained and showed the distribution of trained coefficients; however, it could not quantitatively distinguish the important gene families (Figure S3). Additionally, a random forest classifier was iteratively trained to select the critical features (gene families) for classifying the labeled species. We were able to identify numerous label-associated gene families based on the feature importance values from the trained model (Figure 5A). Based on the feature importance values, we chose the top 20 gene families (Table S2). The random forest classifier was re-trained to test how well it could predict given labels based on the gene families we chose. It is possible that the trained model’s prediction was dependent on the chance because our dataset’s class distribution was excessively biased (Figure 5B). We, iteratively, (1000 times) trained the random forest model with knowledge-based labels and random labels and compared the accuracy scores and area under the receiver operating characteristic curve (AUC) to see if our model predicted the label by chance (Figure 5C). The score and AUC distributions revealed that our trained model was not a result of random chance (Figure 5C). Selected gene families showed informative copy number patterns that were unique to the species showing multiple PAL gene families. There was a clear differentiation between 0 and 1-labeled species in four gene Eggnog clusters, including 37TCT, 37NUR, 37M76, and 37KBC (Figure 5D).

In *Arabidopsis*, the “37TCT” gene family was identified as the transcription factor TT2, which was thought to be involved in flavonoid late metabolism regulation. This gene has also been found to be a positive regulator of stilbene production in *Vitis* [21]. Furthermore, 37KBC is a cytokinin-activating enzyme that works in the direct activation pathway and is annotated as cytokinin riboside 5${}^{\prime }$-monophosphate phosphoribohydrolase. Although there are no direct studies linking resveratrol to this enzyme, the protein rolC has been proposed to have a similar role with the 37KBC gene family in cytokinin metabolism, specifically the conversion of inactive cytokinin glucoside conjugates to active free cytokinins. A previous study found that transforming *Vitis amurensis* with the rolC gene boosted resveratrol production by 3.7 and 11.9 times in two transformed callus cultures [22]. The other two gene families, 37NUR (LRR receptor-like serine threonine-protein kinase) and 37M76 (Tricyclene synthase) have yet to be linked to resveratrol synthesis; nevertheless, the gene count pattern for our labels suggests that they may be involved in resveratrol production in both direct and indirect ways.

## 3. Discussion

The abundance of plant genomes is a result of rapid technological advancements in the field of sequencing. Based on the genomic information, researchers want to determine the gene content of each plant species that has a unique set of benefits for humans. In the case of plant species, however, the genetic knowledge of metabolite synthesis is mainly examined in model species, particularly *Arabidopsis thaliana*. Unless the selected genes are highly conserved across plant species, the reported phenotypes were rarely reproduced when model species genetic knowledge was directly transferred to neighboring species. This is, in part, due to the evolutionary rewiring of each plant genome in response to environmental changes. The gene set that elicits disease resistance to biotic invaders such as bacteria, fungus, and insects, in particular, evolves quickly to optimize energy consumption for growth and defense, often resulting in a gene family with a large number of members or complete absence there of [23]. This is especially true for gene families involved in secondary metabolite synthesis which are downstream of disease recognition genes [24]. Hence, rather than engineering any orthologs in the known pathway, it is vital to understand the crucial factors among them that would distinguish the synthesis of interesting secondary metabolites.

To perform this, a gene family mining technique had to be developed to determine what factors are significant in the creation of known medically and commercially advantageous secondary metabolites of plants. We picked resveratrol as an example for our study because it is a well-studied plant extract with a well-known pathway, and understanding the main genetic components that enhance resveratrol production would be commercially beneficial. We developed a simple semi-supervised machine learning approach for mining the important gene families thought to be involved in the synthesis of resveratrol. Notably, we discovered additional gene families in addition to the well-characterized resveratrol pathway, and a few of them have already been shown to have a direct or indirect effect on resveratrol synthesis.

This strategy can also be utilized to find critical gene components responsible for any secondary metabolites whose pathways have already been studied in the model species. We propose that this straightforward machine learning approach can be used to expand the list of candidate genes for synthetic biology applications such as the production of resveratrol from fermentors and genome editing on the species with a huge biomass production.

## 4. Materials and Methods

### 4.1. Data Preparation

Based on previous research, the resveratrol synthesis pathway was determined [25,26]. The KEGG orthology database was used to assign gene components to the pathway [10]. We used the Eggnog 5.0 database, which assigns an ortholog cluster ID to create the gene family copy number matrix [14]. For this study, we used Viridiplantae’s cluster ID to focus on plants.

### 4.2. Machine Learning and Visualization

The gene family count matrix was hierarchically clustered and heatmap visualized by the Python module seaborn [27]. We used the machine learning approaches implemented by the scikit-learn Python module after assigning labels based on the phenylalanine ammonia lyase (PAL) gene family [28]. The prepared dataset was used to train the ridge and random forest models for feature selection. We attempted to rank the importance in relation to the given labels using the training coefficient and feature importance for ridge and random forest, respectively. As the feature importance values from the random forest model showed better suggestions with regard to the given label, we further tested how well the selected features explained the labels based on the accuracy score together with a pseudo model trained with random labels. The protein sequence alignment of selected gene families was extracted from the Eggnog database API. The phylogenetic trees were constructed with protein domain alignment using ETE Toolkit [29].

## Data Availability

Not applicable.

## Supplementary Materials

The following are available at https://www.mdpi.com/article/10.3390/plants1010000/s1, Figure S1: The gene family count plot of key gene families in resveratrol synthesis pathway, Figure S2: Correlation between resveratrol contents and the ratio of key gene family counts of four plant species, Figure S3: Ridge classification results showing distribution of coefficient (upper left), the accuracy score comparison between true and pseudo model (upper right), and top 20 selection based on coefficient values, Table S1: Dataset for the machine learning, the values are the count of gene families of each species, Table S2: Annotation of selected gene families for resveratrol production.
