# Supplementary material for: The Semi-Supervised Strategy of Machine Learning on the Gene Family Diversity to Unravel Resveratrol Synthesis
Source: Plants (Basel). 2021 Sep 29;10(10):2058. doi: 10.3390/plants10102058 (PMC8538884; doi:10.3390/plants10102058)
Supplement: Supplementary file 1 [file plants-10-02058-s001.zip › Figures_legends.pdf]

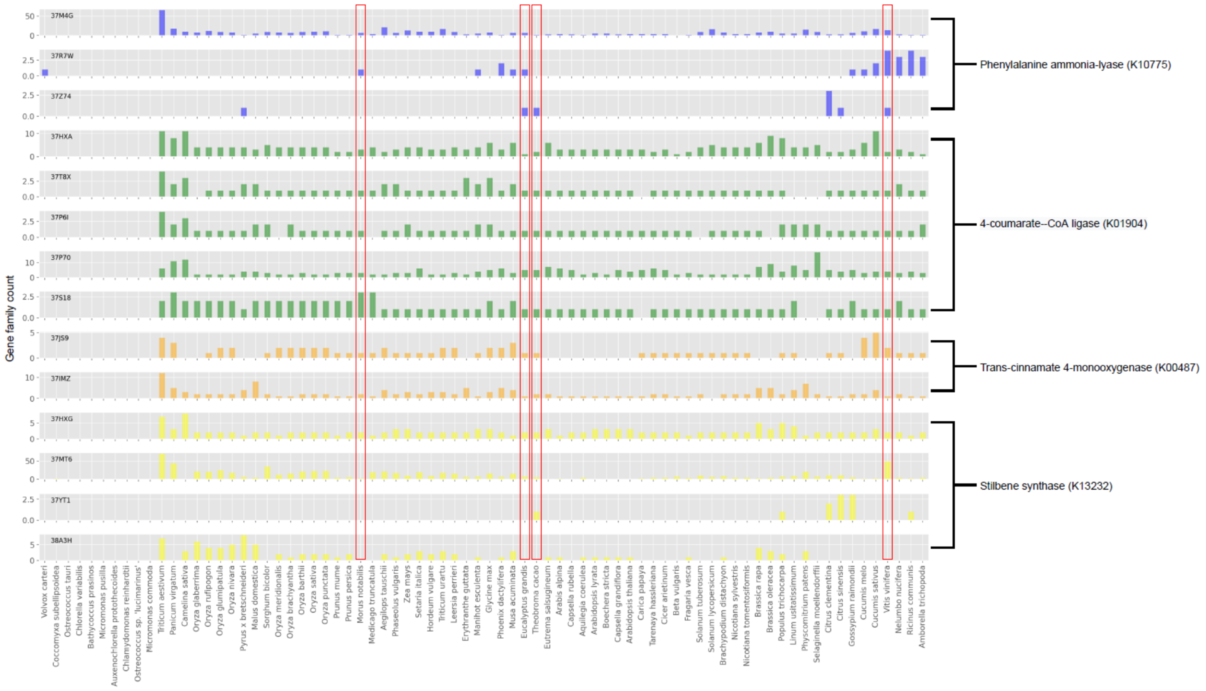

Figure S1. The gene family count plot of key gene families in resveratrol synthesis pathway

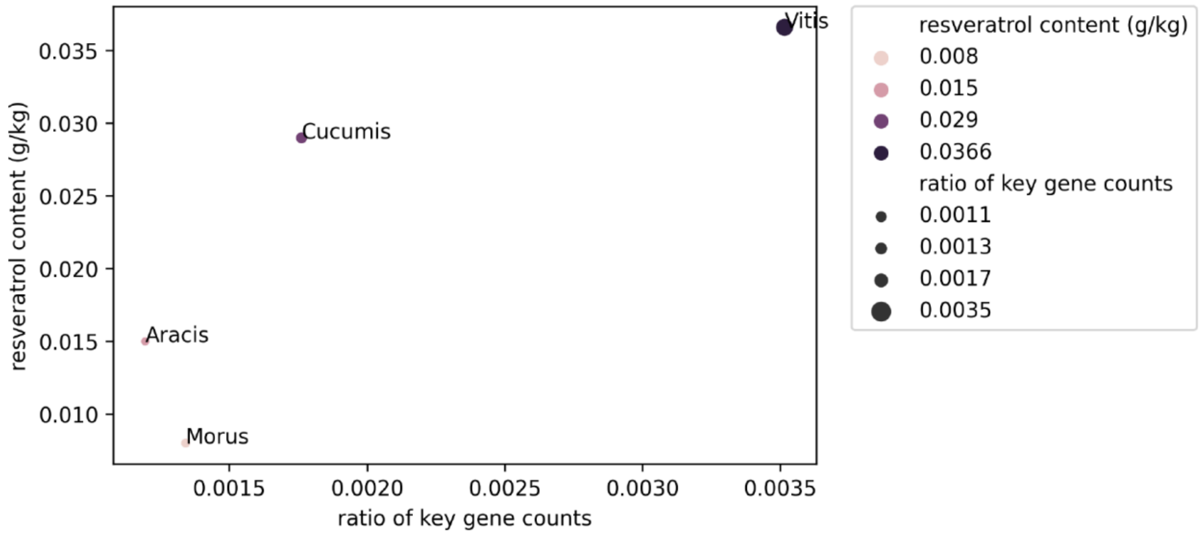

Figure S2. Correlation between resveratrol contents and the ratio of key gene family counts of four plant species.

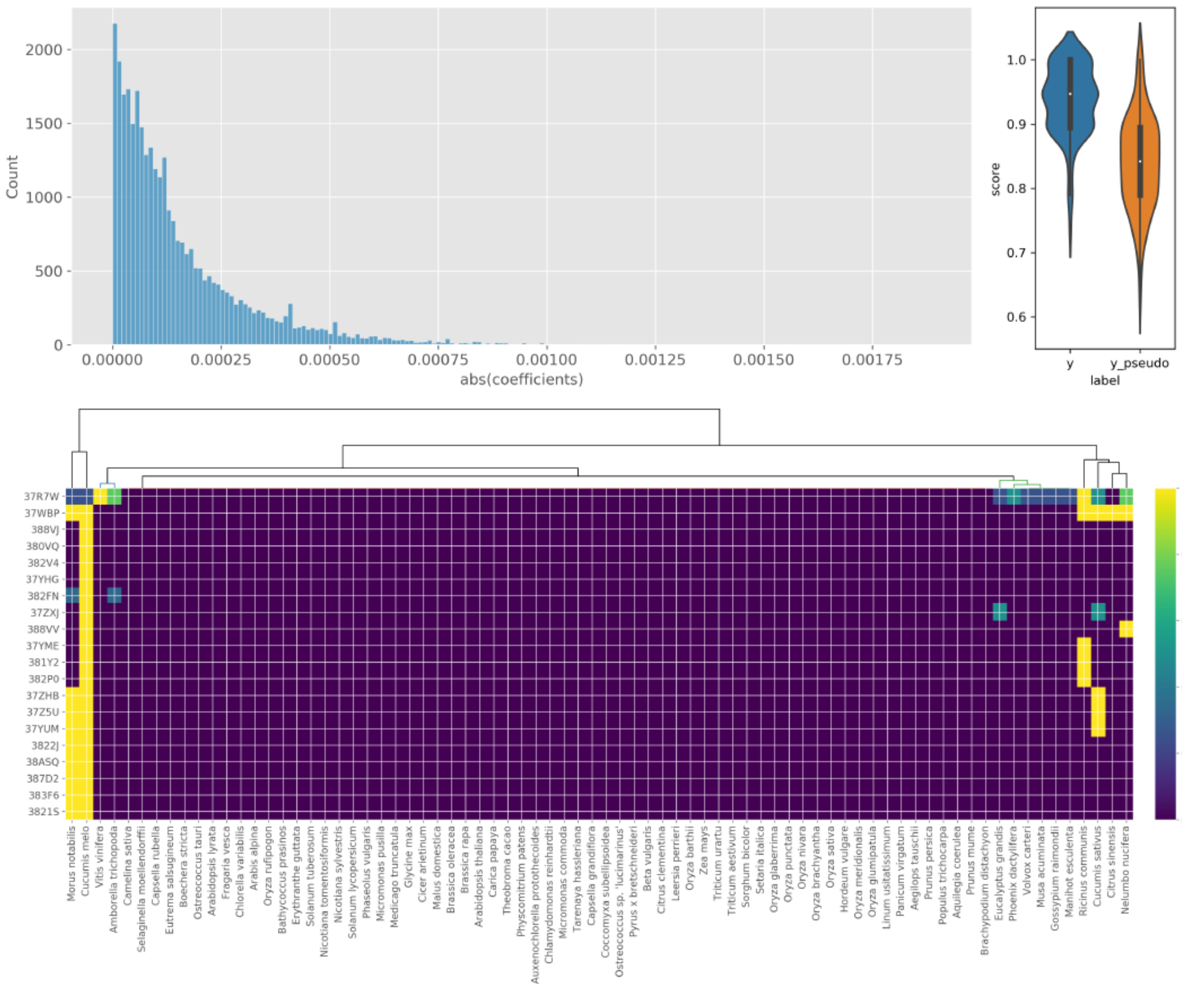

Figure S3. Ridge classification results showing distribution of coefficient (upper left), the accuracy score comparison between true and pseudo model (upper right), and top 20 selection based on coefficient values.
